# Supplementary material for: Splice-Junction-Based Mapping of Alternative Isoforms in the Human Proteome
Source: Cell Rep. Author manuscript; Available in PMC 2020 Jan 15. (PMC6961840; doi:10.1016/j.celrep.2019.11.026)
Supplement: 2 [file NIHMS1546469-supplement-2.zip › DF1/PXD010154/AdrenalGland/AdrenalGland_1_FDXR_TPQICVVGSGPAGFYTAQHLLKG.pdf]

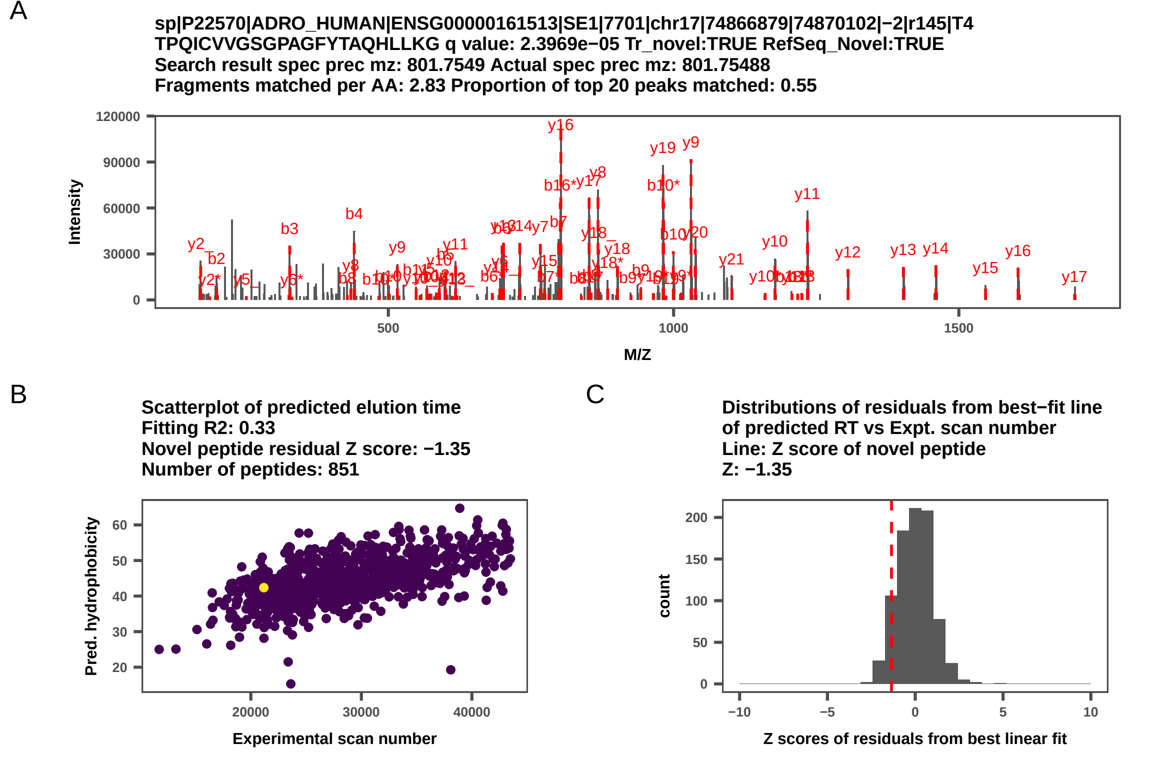
A
sp|P22570|ADRO_HUMAN|ENSG00000161513|SE1|7701|chr17|74866879|74870102|−2|r145|T4
TPQICVVGSGPAGFYTAQHLLKG q value: 2.3969e−05 Tr_novel:TRUE RefSeq_Novel:TRUE
Search result spec prec mz: 801.7549 Actual spec prec mz: 801.75488
Fragments matched per AA: 2.83 Proportion of top 20 peaks matched: 0.55
Intensity
120000
90000
60000
30000
0
500
1000
1500
M/Z
B
Scatterplot of predicted elution time
Fitting R2: 0.33
Novel peptide residual Z score: −1.35
Number of peptides: 851
Pred. hydrophobicity
Experimental scan number
C
Distributions of residuals from best−fit line
of predicted RT vs Expt. scan number
Line: Z score of novel peptide
Z: −1.35
count
Z scores of residuals from best linear fit
